# SARS-CoV-2 percent positivity and risk factors among people with HIV at an urban academic medical center

**DOI:** 10.1371/journal.pone.0254994

**Published:** 2021-07-21

**Authors:** Eleanor E. Friedman, Samantha A. Devlin, Moira C. McNulty, Jessica P. Ridgway

**Affiliations:** 1 Chicago Center for HIV Elimination, Chicago, IL, United States of America; 2 Department of Medicine, University of Chicago, Chicago, IL, United States of America; Rush University, UNITED STATES

## Abstract

Since the onset of the COVID-19 pandemic, it has been unclear how vulnerable people with HIV (PwH) are to SARS-CoV-2 infection. We sought to determine if PwH are more likely to test positive for SARS-CoV-2 than people without HIV, and to identify risk factors associated with SARS-CoV-2 positivity among PwH. We conducted a cross-sectional study in which we collected electronic medical record data for all patients who underwent SARS-CoV-2 PCR testing at an academic medical center. Presence of HIV and other chronic diseases were based on the presence of ICD-10 diagnosis codes. We calculated the percent positivity for SARS-CoV-2 among PwH and among people without HIV. Among PwH, we compared demographic factors, comorbidities, HIV viral load, CD4 T-cell count, and antiretroviral therapy (ART) regimens between those who tested positive for SARS-CoV-2 and those who tested negative. Comparisons were made using chi squared tests or Wilcoxon rank sum tests. Multivariate models were created using logistic regression. Among 69,763 people tested for SARS-CoV-2, 0.6% (431) were PwH. PwH were not significantly more likely to test positive for SARS-CoV-2 than people without HIV (7.2% (31/431) vs 8.4% (5820/69763), p = 0.35), but were more likely to be younger, Black, and male (p-values < .0001). There were no significant differences in HIV clinical factors, chronic diseases, or ART regimens among PwH testing positive for SARS-CoV-2 versus those testing negative. In our sample, PwH were not more likely to contract SARS-CoV-2, despite being more likely to be members of demographic groups known to be at higher risk for infection. Differences between PwH who tested positive for SARS-CoV-2 and those who tested negative were only seen in Hispanic/Latino ethnicity (non-Hispanic or Latino vs unknown Hispanic or Latino ethnicity (OR 0.2 95% CI (0.6, 0.9)) and site of testing(inpatient vs outpatient OR 3.1 95% CI (1.3, 7.4)).

## Introduction

Coronavirus disease 2019 (COVID-2019), caused by severe acute respiratory syndrome coronavirus 2 (SARS-CoV-2), remains a significant problem throughout the world, with more than 29 million confirmed cases and over 533,000 deaths in the United States as of March 16^th^ 2021 [1]. The COVID-19 pandemic has disproportionately affected certain populations, including older people (≥60 years) and those with underlying chronic health conditions such as diabetes, hypertension, and obesity [2]. Among persons with immune suppressed conditions, such as human immunodeficiency virus (HIV), the level of vulnerability to COVID-19 is unclear [3]. The risk of COVID-19 acquisition among people living with HIV (PwH) is complicated by varying degrees of immunosuppression, multimorbidity, and the possible effect of antiretroviral (ART) drugs [4].

Current research has indicated similar risk of SARS-CoV-2 infection between PwH and those without HIV. The incidence or percent positivity of SARS-CoV-2 among PwH, particularly those who are virologically suppressed, appears similar to—if not lower than—that among HIV negative people [4–10]. During an ongoing pandemic, especially one that has a high percentage of asymptomatic or mildly symptomatic infections, determining true risk factors as opposed to those that simply increase disease severity is complex. Studies that have reported on the health outcomes of hospitalized COVID-19 patients are subject to selection bias, producing risk factors that may not exist within the general population or mistakenly conflating risk of SARS-CoV-2 infection with risk of COVID-19 disease severity [11]. Risk factors that increase susceptibility to SARS-CoV-2 in the general population also exist among PwH. Nearly half (40%) of the approximately 1.2 million PwH in the United States [12] are older than 50 years [13]. Additionally, many PwH have significant underlying health conditions [14] that can increase the risk of COVID-19 disease severity and the risk of contracting SARS-CoV-2 infection. Several studies have shown that the majority of PwH who contracted SARS-CoV-2 and were subsequently hospitalized or died from COVID-19 had multiple underlying comorbidities, despite being on ART with good virologic control [4, 5, 15–18].

Among PwH, potential biological risk factors for SARS-CoV-2 infection include HIV-related clinical markers such as viral suppression and CD4 T cell count, as well as ART drug class [19]. Although low CD4 counts have not been associated with SARS-CoV-2 incidence, immunosuppression appears to affect COVID-19 disease severity among PwH [16, 20]. Recent ART initiation or poor ART adherence may increase the risk for SARS-CoV-2 acquisition and COVID-19 disease severity among PwH [21, 22]. Molecular studies have suggested that some nucleoside/nucleotide reverse transcriptase inhibitors (NRTIs), including tenofovir disoproxil fumarate (TDF), tenofovir alafenamide (TAF), abacavir (ABC), and lamivudine (3TC), may protect against SARS-CoV-2 acquisition [6, 23, 24]. However, most studies have found no association between antiretroviral drug regimen and SARS-CoV-2 infection or COVID-19 disease severity [5, 8, 10, 19, 20, 25] PwH have multiple reasons to be concerned that they are at greater risk of infection, morbidity, and mortality from SARS-CoV-2. Biological factors, co-morbidities, and social, economic, and structural factors. may all increase the risk of COVID-19 disease severity among PwH. It is virtually impossible to pinpoint which of these sources is most responsible for their increased risk, as each element has a varying but significant impact on PwH. Of note, most studies to date among PwH have examined SARS-CoV-2 incidence or percent positivity among severe or hospitalized COVID-19 cases, settings where mild or asymptomatic cases of SARS-CoV-2 infection may have been missed [7, 8, 10, 19]. Few studies have examined risk factors for SARS-CoV-2 infection among PwH who were identified via community-based testing programs.

A great deal of research has been devoted to determining if COVID-19 is more severe among PwH than in the general population. Some studies have focused on describing the severity and clinical outcomes related to COVID-19 for hospitalized PwH [4, 9, 10, 19], while others have compared adverse COVID-19 outcomes between PwH and people without HIV. Some of these studies have found no difference in health outcomes between the populations but a higher crude COVID-19 mortality rate in PwH [16], and others finding both poorer outcomes and a higher COVID-19 mortality rate in PwH than those without HIV [26]. While this is of extreme importance to both researchers as well as the PWH community, it is also important to determine if PwH are uniquely susceptible to SARS-CoV-2 infection, either directly due to infection and treatment related reasons, or indirectly related to the socio-demographics of PwH.

We examined cases of SARS-CoV-2 infection among PwH from a large hospital and community SARS-CoV-2 testing program in Chicago, IL. This testing population included symptomatic (severe and mild cases) COVID-19 cases as well as asymptomatic cases of SARS-CoV-2 infection. We sought to determine if there was a difference in the percent positivity of SARS-CoV-2 between PwH and HIV negative people in this population. We also sought to identify any risk factors that distinguish PwH who tested positive for SARS-CoV-2 from PwH who tested negative. In particular, we examined demographic factors, ART regimens, comorbidities, and HIV clinical factors among PwH.

## Materials and methods

We collected data for all patients tested for SARS CoV-2 RNA PCR between April 10^th^ 2020 and September 30^th^ 2020 at University of Chicago Medicine (UCM). UCM is a large urban academic medical center on the south side of Chicago, serving a diverse area with many economically disadvantaged neighborhoods. UCM is also one of the main HIV care providers on Chicago’s south side. UCM has a large SARS-CoV-2 testing program, providing testing for UCM patients, healthcare providers, and non-affiliated community members. This testing program included testing at the emergency department and inpatient departments, drive-thru testing, and pre-procedural testing before surgical procedures. This study was reviewed by the University of Chicago Institutional Review Board and determined to be exempt as all data used in this study were de-identified. As an exempt study, no consent from subjects was sought.

Using a cross-sectional study design, all information was drawn from the patient’s electronic medical record. Deidentified data were provided by the Center for Research Informatics from the Clinical Research Data Warehouse at the UCM All participants were tested for SARS COV-2 RNA using Cepheid Xpert Xpress PCR or Roche cobas SARS-CoV-2 PCR. If a participant tested both negative and positive, they were included only among the positives. In total 460 positive persons previously tested as negative. For persons with multiple tests with the same result (either negative or positive), information from the first test date was used.

Information on HIV ART was drawn both from most recent prescription drugs ordered and administered as well as current medications listed in the patient’s clinical notes. Categorization of drug types and regimens were based on prior literature that suggested more favorable outcomes or even prevention of COVID-19 among particular HIV ART classes [6]. HIV status, the presence of chronic conditions, and if SARS-CoV-2 testing was conducted pre-procedurally were determined by the presence of ICD-10-CM codes. Persons without the presence of these codes were considered negative for HIV or for the chronic condition. Chronic conditions were chosen that have been previously found to be associated with severe COVID-19 disease [15, 16, 20]. HIV laboratory measurements were categorized as follows: CD4 T cell values below 350 cells/mm3 were considered low, whereas viral load results below 200 copies per milliliter were considered virally suppressed. Race, ethnicity, and sex information were drawn from structured fields provided in the electronic medical record. Due to the homogeneity of our patient population, race was collapsed into African American/Black, white, other, and unknown. Other race included the following categories: Asian/Mideast Indian, more than one race, American Indian or Alaskan Native, and Native Hawaiian/Other. Unknown race included the following categories: Patient declined and unknown. Insurance information was categorized into Medicare, Medicaid, private, and unknown. Location of SARS-CoV-2 testing (inpatient, outpatient, or emergency department) was drawn from the testing encounter record.

Categorical variables were tested for significance using chi-square or Fisher’s exact tests and p-values. Continuous variables were examined for normality, and if not normal were tested with Wilcoxon rank sum test and p-value. Associations between SARS-CoV-2 and medical or demographic factors were assessed using logistic regression with odds ratios and 95% confidence intervals (OR, 95% CI). All data analysis was done using SAS version 9.4 (Cary, North Carolina).

## Results

In total 69,763 persons were tested for SARS-CoV-2 at the University of Chicago Medicine (UCM) between April 10^th^ 2020 and September 30^th^ 2020. Among those tested for SARS-CoV-2, we identified 431 PwH. Among PwH, 31 (7.2% 95% CI (4.8%, 9.6%)) tested positive for SARS-CoV-2. This proportion of positive for SARS-CoV-2 was not significantly different than that seen in the HIV negative population (8.4% 95% CI (8.2%, 8.6%)) (0.37 p-value). Significant demographic and insurance differences were seen between PwH and HIV negative persons. However, these differences reflect the epidemiology of the HIV epidemic in Chicago, which has disproportionately impacted certain communities, as well as the primary sources of insurance for PwH (Table 1) [27]. PwH tested for SARS-CoV-2 were more likely to be young (both 26–45 years of age and 46–65 years of age vs over 65 (OR 4.3 95% CI (3.0, 6.1)) and OR 4.2 95% CI (3.0, 6.0)), African American/Black (African American/Black vs white OR 7.4 95% CI (5.1,10.7)) and male (male sex vs female sex OR 3.3 95% CI (2.7, 4.1)). PwH were less likely to be Hispanic/Latino (Hispanic/Latino vs not Hispanic/Latino OR 0.3 95% CI (0.2, 0.6)) than other persons tested for COVID-19. PwH were more likely to have Medicaid or Medicare insurance (Medicaid vs private OR 4.4 95% CI (3.4, 5.7) Medicare vs private OR 2.9 95% CI (2.2, 3.9)). PwH also differed from HIV negative persons in where they were tested for SARS-CoV-2. PwH were far more likely to have tested in emergency or inpatient settings than in outpatient settings (emergency vs outpatient OR 6.0 95% CI (4.8, 7.4), inpatient vs outpatient OR 6.6 95% CI (5.1, 8.4)), as well as more likely to be tested pre-procedurally (pre-procedural testing yes vs no OR 1.6 95% CI (1.2, 2.1)). PwH were also significantly more likely to have one or more of the chronic diseases we examined (Table 1).

**Table 1:** Baseline demographic and disease characteristics of all persons tested for SARS-CoV-2 (N = 69763).

	PwH (N = 431)	HIV- or undiagnosed (N = 69332)	P value
Age, median (IQR)	47 (32–58)	58 (25–71)	0.09∞
Sex			
Male	307 (71.2%)	29538 (42.6%)	< .0001
Female	124 (28.8%)	39775 (57.3%)	
Unknown	0 (0.0%)	39 (0.06%)	
Race			
Black	376 (87.2%)8	25245 (36.4%)	< .0001
White	(1.86%)	3010 (4.3%)	
Other	31 (7.19%)	15444 (22.3%)	
Unknown	16 (3.7%)	25624 (37.0%)	
Ethnicity			
Hispanic	10 (2.3%)	3060 (4.4%)	< .0001
Non-Hispanic	409 (94.9%)	40769 (58.8%)	
Unknown	12 (2.8%)	25503 (36.8%)	
Insurance			
Medicaid	212 (49.2%)	15241 (22.0%)	< .0001
Medicare	116 (26.9%)	12552 (18.1%)	
Private	75 (17.4%)	23760 (34.3%)	
Unknown	28 (6.5%)	17813 (25.7%)	
COVID-19 results			
Negative	400 (92.8%)	63509 (91.6%)	0.37
Positive	31 (7.2%)	5820 (8.4%)	
Testing location			
Emergency	155 (36.0%)	8485 (12.2%)	< .0001
Inpatient	107 (24.8%)	5326 (7.7%)	
Outpatient	169 (39.2%)	55191 (79.6%)	
Unknown	0 (0.0%)	330 (0.5%)	
Pre-procedural testing			
Yes	49 (11.4%)	5264 (7.6%)	0.003
No	382 (88.6%)	64068 (92.1%)	
Chronic conditions			
Diabetes	61 (14.2%)	6262 (9.0%)	< .0001
Hypertension	206 (47.8%)	13708 (19.8%)	< .0001
Congestive heart failure	18 (4.2%)	670 (1.0%)	< .0001
Coronary artery disease	44 (10.2%)	3285 (4.7%)	< .0001
Malignant cancer	51 (11.8%)	5613 (8.1%)	0.005
COPD±	49 (11.4%)	1194 (3.3%)	< .0001
Asthma	72 (16.7%)	4386 (6.3%)	< .0001
Sleep apnea	29 (6.7%)	3420 (4.9%)	0.09
Hepatitis C	18 (4.2%)	293 (0.4%)	< .0001
Hepatitis B	10 (2.3%)	72 (0.1%)	< .0001
Renal disease	77 (17.9%)	3840 (5.5%)	< .0001
Morbid obesity	49 (11.4%)	5907 (8.5%)	0.04
Any chronic disease	277 (64.3%)	22075 (31.8%)	< .0001

P values are χ^2^ unless noted.

*Fisher’s exact p-value.

∞ Wilcoxon two sample test of medians p-value.

When only PwH were examined, few significant differences in demographic factors, chronic conditions, HIV clinical factors, ART usage, or ART regimen were seen (Table 2). The only significant demographic factor seen was that PwH who tested positive for SARS-CoV-2 were less likely to be non-Hispanic or Latino as compared to being of unknown Hispanic or Latino ethnicity (OR 0.2 95% CI (0.6, 0.9)). A sensitivity analysis that modeled other cut points for viral suppression and low CD4 T cell counts resulted in similar non-significant results. Testing location was significantly different between those who were SARS-CoV-2 positive, with inpatient testing more likely (inpatient vs outpatient OR 3.1 95% CI (1.3, 7.4)).

**Table 2:** Baseline demographic characteristics of PwH tested for SARS-CoV-2.

	PwH (N = 431 (100%))	PwH COVID- (N = 400 (92.8%))	PwH COVID+ N = 31 (7.2%)	P-value
Age, median (IQR)	47 (32–58)	46 (32–58)	49 (34–59)	0.56∞
Sex				
Male	307 (71.2%)	284 (71.0%)	23 (74.2%)	0.71
Female	124 (28.8%)	116 (29.0%)	8 (25.8%)	
Race				
Black	376 (87.2%)	348 (87.0%)	28 (90.3%)	0.63*
White	8 (1.86%)	30 (7.5%)	1 (3.2%)	
Other	31 (7.19%)	8 (2.0%)	0 (0.0%)	
Unknown	16 (3.7%)	14 (3.5%)	2 (6.5%)	
Ethnicity				
Hispanic	10 (2.3%)	10 (2.5%)	0 (0.0%)	0.01*
Non-Hispanic	12 (2.8%)	9 (2.3%)	3 (9.7%)	
Unknown	409 (94.9%)	381 (95.3%)	28 (90.3%)	
Insurance				
Medicaid	212 (49.2%)	200 (50.0%)	12 (38.7%)	0.47*
Medicare	116 (26.9%)	104 (26.0%)	12 (38.7%)	
Private	75 (17.4%)	70 (17.5%)	5 (16.1%)	
Unknown	28 (6.5%)	26 (6.5%)	2 (6.5%)	
Testing location				
Emergency	155 (36.0%)	149 (37.3%)	6 (19.4%)	0.001
Inpatient	107 (24.8%)	91 (22.8%)	16 (51.6%)	
Outpatient	169 (39.2%)	160 (40.0%)	9 (29.0%)	
Unknown	0 (0.0%)	0 (0.0%)	0 (0.0%)	
Pre-procedural testing				
Yes	49 (11.4%)	48 (12.0%)	1 (3.2%)	0.23*
No	382 (88.6%)	352 (88.0%)	30 (96.8%)	
Most recent VL				
>200 copies per ml	95 (22.0%)	90 (22.5%)	5 (16.1%)	0.47
≤200 copies per ml	247 (57.3%)	226 (56.5%)	21 (67.7%)	
Missing	89 (20.7%)	84 (21.0%)	5 (16.1%)	
Most recent CD4				
≥ 350	197 (45.7%)	186 (46.5%)	11 (35.5%)	0.47
<350	133 (30.9%)	121 (30.3%)	12 (38.7%)	
Missing	101 (23.4%)	93 (23.3%)	8 (25.8%)	
ART regimen				
ABC/3TC	24 (5.6%)	23 (5.8%)	1 (3.2%)	0.99*
FTC/TDF	29 (6.7%)	27 (6.8%)	2 (6.5%)	0.99*
FTC/TAF	246 (57.1%)	228 (57.0%)	18 (58.1%)	0.91
Any INSTI	286 (66.4%)	266 (66.5%)	20 (64.5%)	0.82
Any NRTI	303 (70.3%)	281 (70.3%)	22 (71.0%)	0.93
Any NNRTI	39 (9.1%)	35 (8.8%)	4 (12.9%)	0.51*
Any PK booster	84 (19.5%)	77 (19.3%)	7 (25.6%)	0.65
Any PI	50 (11.6%)	45 (11.3%)	5 (16.1%)	0.41
Any ART	325 (75.4%)	301 (75.3%)	24 (77.4%)	0.79
Missing	106 (24.6%)	83 (25.5%)	7 (22.6%)	NA
Chronic conditions				
Diabetes	61 (14.2%)	57 (14.3%)	4 (12.9%)	0.99*
Hypertension	206 (47.8%)	195 (48.8%)	11 (35.5%)	0.15
Congestive heart failure	18 (4.2%)	15 (3.8%)	3 (9.7%)	0.13*
Coronary artery disease	44 (10.2%)	42 (10.5%)	2 (6.5%)	0.75*
Malignant cancer	51 (11.8%)	49 (12.3%)	2 (6.5%)	0.56*
COPD±	49 (11.4%)	46 (11.5%)	3 (9.7%)	0.99*
Asthma	72 (16.7%)	69 (17.3%)	3 (9.7%)	0.45*
Sleep apnea	29 (6.7%)	28 (7.0%)	1 (3.2%)	0.71*
Hepatitis C	18 (4.2%)	17 (4.3%)	1 (3.2%)	0.99*
Hepatitis B	10 (2.3%)	9 (2.3%)	1 (3.2%)	0.53*
Renal disease	77 (17.9%)	72 (19.6%)	5 (16.1%)	0.79
Morbid obesity	49 (11.4%)	45 (11.3%)	4 (12.9%)	0.77*
**Any chronic disease**	**277 (64.3%)**	**259 (64.8%)**	**18 (58.1%)**	**0.45**

P values are χ^2^ unless noted.

*Fisher’s exact p-value.

∞ Wilcoxon two sample test of medians p-value.

±Chronic Obstructive Pulmonary Disease.

## Discussion

In this study, we describe a population of PwH in Chicago, IL who were tested for SARS-CoV-2. The vast majority of PwH who were tested for SARS-CoV-2 tested negative. The SARS-CoV-2 percent positivity rate among PwH was slightly lower than that of HIV-negative or undiagnosed individuals in the general population, corroborating previous studies that report no significant difference in the incidence or positivity of and risk for SARS-CoV-2 infection among PwH [4–10, 28].

The PwH in our sample were mostly Black, young, and male, reflecting a population on the south side of Chicago that is particularly vulnerable to COVID-19 due to social determinants of health related to both poverty and race [29]. Overall, the dual burden of HIV and COVID-19 has disproportionately impacted communities of color throughout the United States [30]. Additionally, we found that PwH in this study were more likely to have chronic conditions which have been associated with more severe COVID-19 disease, a finding seen in other studies examining PwH and COVID-19 [16, 17, 26, 28]. Similar to our findings, Hadi et al. demonstrated that in comparison to people without HIV, PwH diagnosed with COVID-19 were more likely to be African American and male and have concurrent conditions such as hypertension, diabetes, and chronic kidney disease [18]. PWH were also more likely to have Medicaid or Medicare insurance rather than private insurance, which reflects the high utilization of public insurance by PWH in our clinic. While our findings agree with the majority that indicated no increased risk for SARS-CoV-2 infection for PwH as compared to HIV negative people, unlike other studies (for instance, Tesoriero et al.) our results did not differ between unadjusted and adjusted analyses, despite the fact that PwH had more comorbidities [26]. There are several possible reasons for the lack of increased positivity of SARS-CoV-2 even in unadjusted analyses in this study, such as younger age of participants and potential fear of COVID-19 exposure in an already vulnerable group of patients.

PwH were younger than those who were HIV negative, which may have resulted in fewer positive SARS CoV-2 RNA PCR tests among PwH early in the epidemic when testing and infections among older persons predominated [31]. It is possible that the absence of increased SARS-CoV-2 infection rates in PwH stems from the balance between increased demographic and chronic disease risks and the reduced age-related risk. Additionally, PwH may also have been more concerned about COVID-19 and thus more likely to seek testing, resulting in a selection bias for PwH who were ‘worried well’. This concern may have also caused PwH to adhere to social distancing and take additional precautions to prevent SARS-CoV-2 infection than HIV-negative persons. This increased testing among the worried well was seen in a study conducted by Park et al. who examined testing among PwH in the veterans aging cohort [32]. Notably, although more testing was seen in Park et al., the percent positivity was not different among PwH and HIV-negative persons, confirming our study findings. Theoretically, if well people are concerned and therefore testing more often, it could bias the results of SARS-CoV-2 positivity toward the null, reducing or ameliorating a possible increased risk of SARS-CoV-2 among PwH.

Many previous studies examining SARS-CoV-2 incidence or percent positivity in PwH examined only symptomatic COVID-19 cases who underwent SARS CoV-2 PCR testing [7, 8, 10, 19]. However, our study was performed in the setting of a combined hospital and community-based testing program. In addition to testing patients with symptoms of COVID-19, our hospital also detected positive cases of SARS-CoV-2 among asymptomatic patients who were being tested prior to outpatient routine procedures or during outpatient visits. The inclusion of less severe cases in our sample lends further proof to the theory that SARS-CoV-2 infection is not more common among PwH. Additionally, we used confirmed SARS-CoV-2 negative cases among PwH as our comparison group, unlike some previous studies which did not examine PwH who tested negative for SARS-CoV-2 [4, 7–9, 16, 20].

In accordance with some previous research findings, ART regimen had no significant impact on the SARS-CoV-2 percent positivity among PwH in our sample, suggesting a lack of any protective effect against SARS-CoV-2 infection [5, 8, 10, 19, 20, 25]. These findings are in contrast to those of Del Amo et al. who found that the risk of infection and related hospitalization was lower in patients receiving TDF/FTC than those receiving other regimens [6]. Similarly, there were no differences between the comorbidities in PwH with differing SARS-CoV-2 infection status. Despite our finding that PwH are not more likely to be SARS-CoV-2 positive compared to people without HIV, we did not directly compare the severity of COVID-19 outcomes between the two populations. The difference in testing location between PwH and HIV-negative persons, with PwH more likely to receive testing at either the emergency room or inpatient rather than outpatient testing, may well suggest that PwH with COVID-19 had more severe symptoms than HIV-negative persons. It is possible that had we conducted an extensive hand review of the data, we would have confirmed that the PwH we identified had worse outcomes or more severe disease after diagnosis with SARS-CoV-2 infection compared to the HIV-negative population, as has been found in some studies examining COVID-19 disease progression among PwH [26, 28].

Although our overall sample of persons, as well as our population of PwH who tested for SARS-CoV-2 were large, we identified a limited number of PwH who were positive for SARS-CoV-2. This small sample size may have prevented us from detecting differences between PwH who tested negative for SARS-CoV-2 and PwH who tested positive, particularly related to varying ART regimens and comorbidities. Larger prospective studies should examine these factors to confirm that these are not potential risk factors for SARS-CoV-2 percent positivity among PwH.

Although most recent viral load, CD4 cell count, and ART regimen were used in this analysis, our information on these outcomes was only drawn from our hospital’s medical records, biasing our results to represent patients seen in our HIV clinic and resulting in approximately 25% missing data in these areas. The majority of patients in this sample were on highly active antiretroviral therapy, with HIV viral suppression and good HIV virologic control. Therefore, these findings may not apply to a population with poorly controlled HIV or AIDS. As PwH disproportionately suffer from underlying comorbidities, it is difficult to compare the effect of underlying chronic diseases among PwH, who already have a higher baseline of comorbidities compared to the general population [4, 5, 16, 17]. Finally, a limitation of using an EMR-based data source was the lack of data on socioeconomic factors, employment information, and contact with confirmed COVID-19 cases. These factors could not be examined for associations with SARS-CoV-2 percent positivity and may explain why we found no increase in the proportion of positive SARS-CoV-2 cases among PwH in this study.

## Conclusions

Despite these limitations, our findings provide further evidence that PwH appear to have similar rates of SARS-CoV-2 percent positivity as HIV-negative people within the general population, and there were no obvious risk factors among PwH that increase their chances of testing positive for SARS-CoV-2 in this sample.
